# Supplementary material for: Laminar microcircuitry of visual cortex producing attention-associated electric fields
Source: eLife. 2022 Jan 28;11:e72139. doi: 10.7554/eLife.72139 (PMC8846592; doi:10.7554/eLife.72139)
Supplement: Supplementary file 1. [file elife-72139-supp1.docx]

| *Monkey* | *tASK* | *rECORDING tYPE* | *rECORDING sYSTEM* | *rECORDING eLECTRODES* |
| --- | --- | --- | --- | --- |
| p | **8-Item T/L Search** | **10/20 EEG** | **Plexon MAP** | **Custom Skull-embedded 10/20 System** |
| z | **8-Item Color Pop-Out Search** | **10/20 EEG** | **Plexon MAP** | **Custom Skull-embedded 10/20 System** |
| ca | **6-Item Color Pop-Out Search** | **Laminar V4 + Extracortical Signal** | **TDT PZ5 + RZ2** | **Custom 32-channel Linear Plexon S-Probe** |
| he | **6-Item Color Pop-Out Search** | **Laminar V4 + Extracortical Signal** | **TDT PZ5 + RZ2** | **Custom 32-channel Linear Plexon S-Probe** |

**Supplementary File 1.** Summary detailing the task and recording information for each of the four monkeys used in this study.
